# Solid Lipid–Polymer Hybrid Nanoplatform for Topical Delivery of siRNA: In Vitro Biological Activity and Permeation Studies

**DOI:** 10.3390/jfb14070374

**Published:** 2023-07-17

**Authors:** Margarete Moreno de Araujo, Livia Neves Borgheti-Cardoso, Fabíola Garcia Praça, Priscyla Daniely Marcato, Maria Vitória Lopes Badra Bentley

**Affiliations:** School of Pharmaceutical Sciences of Ribeirao Preto, University of São Paulo, Av. do Café, s/n, Ribeirão Preto 14040-903, SP, Brazil; margaretemoreno@gmail.com (M.M.d.A.); pmarcato@fcfrp.usp.br (P.D.M.)

**Keywords:** solid lipid–polymer hybrid nanoparticles, siRNA, nucleic acid, gene silencing, topical delivery, skin penetration

## Abstract

Small interfering RNA (siRNA) molecules have limited transfection efficiency and stability, necessitating the use of delivery systems to be effective in gene knockdown therapies. In this regard, lipid–polymeric nanocarriers have emerged as a promising class of nanoparticles for siRNA delivery, particularly for topical applications. We proposed the use of solid lipid–polymer hybrid nanoparticles (SLPHNs) as topical delivery systems for siRNA. This approach was evaluated by assessing the ability of SLPHNs–siRNA complexes to internalize siRNA molecules and both to penetrate skin layers in vitro and induce gene knocking down in a skin cell line. The SLPHNs were formed by a specific composition of solid lipids, a surfactant polymer as a dispersive agent, and a cationic polymer as a complexing agent for siRNA. The optimized nanocarriers exhibited a spherical shape with a smooth surface. The average diameter of the nanoparticles was found to be 200 nm, and the zeta potential was measured to be +20 mV. Furthermore, these nanocarriers demonstrated excellent stability when stored at 4 °C over a period of 90 days. In vitro and in vivo permeation studies showed that SLPHNs increased the cutaneous penetration of fluorescent-labeled siRNA, which reached deeper skin layers. Efficacy studies were conducted on keratinocytes and fibroblasts, showing that SLPHNs maintained cell viability and high cellular uptake. Furthermore, SLPHNs complexed with siRNA against Firefly luciferase (siLuc) reduced luciferase expression, proving the efficacy of this nanocarrier in providing adequate intracellular release of siRNA for silencing specific genes. Based on these results, the developed carriers are promising siRNA delivery systems for skin disease therapy.

## 1. Introduction

The use of RNA interference (RNAi) is a promising therapeutic approach to modulate or inhibit endogenous gene expression [1,2,3,4,5] through synthetic small interfering nucleic acid (siRNA) therapeutics [6,7]. RNAi is mediated by double-stranded siRNA molecules consisting of 21–27 base pairs and has demonstrated a remarkable ability to effectively suppress the expression of specific genes [8]. However, one of the major challenges in siRNA therapy is the effective transfer of (naked) nucleic acid across cell membranes due to the anionic charge of this molecule as well as its poor stability in biological environments [9,10,11].

The administration route is another issue that must be considered for the success of the siRNA clinical approach. Topical administration of siRNA targeting genes involved in several skin disorders offers a fresh and innovative therapeutic strategy for addressing inherited skin conditions, viral infections, skin cancer, and atopic dermatitis [10,12,13]. However, the clinical application of siRNA into the skin is limited by the efficient barrier properties of the skin provided by the stratum corneum (SC) [14]. To surmount the inherent obstacles associated with the delivery of siRNA to the skin, nanoparticles have been devised [2,12,13,14,15,16,17]. Through these nanotechnologies, high penetration rates of siRNA into the skin can be achieved by multiple pathways, including intercellular, transcellular, and appendage pathways. For instance, lipid nanoparticles can interact with intercellular lipids in the SC, which is the outermost layer of the skin, and disrupt the organization of the SC lipids, creating temporary gaps that allow siRNA penetration. This pathway involves siRNA release from the nanoparticles and its diffusion among corneocytes (flat, dead skin cells) through the lipid-rich intercellular spaces. siRNA can also penetrate corneocytes via a transcellular route. Cellular uptake of siRNA-loaded nanoparticles occurs through endocytosis or other internalization mechanisms. Once inside the cells, nanoparticles can release their cargo (siRNA) into the cytoplasm, where silencing of target genes takes place. Moreover, skin appendages such as hair follicles and sweat glands provide an alternate route for penetration. siRNA carried by nanoparticles can enter hair follicles or sweat ducts and then travel through the surrounding skin layers to reach the target cells [2].

Several nanotechnology platforms have been studied for topical delivery of siRNA, including nanostructured delivery systems that can be produced using different compounds. Among them, polyethylenimine (PEI) has shown remarkable efficacy in delivering siRNA due to its amino groups that can be protonated at physiological pH and its strong buffering capacity [18,19]. Nevertheless, the formation of PEI/siRNA complexes frequently leads to elevated cytotoxicity [12,20]. Thus, the clinical application of siRNA requires the use of effective and biodegradable delivery systems that simultaneously exhibit low cytotoxicity. In the present work, we developed a solid lipid–polymer hybrid nanoparticle (SLPHN) for the effective delivery of siRNA. The SLPHNs combine appropriate siRNA delivery characteristics of PEI and solid lipid nanoparticles (SLN), which have high biocompatibility and bioavailability, controlled drug release, physical stability, and protection of incorporated labile drugs from degradation, while their production process is easily scalable [21,22,23,24].

Our optimized SLPHNs consist of Compritol^®^ 888 ATO as a structural lipid, PEI as a cationic agent, and poloxamer 188 as a surfactant. Several combinations of these components were investigated in order to select the best composition that provided small particles of homogeneous sizes (polydispersity index) and positive charge surface (zeta potential). Then, the optimized SLPHN samples were characterized regarding their physicochemical properties by DLS, NTA, and AFM. The efficiency of the siRNA–SLPHN complex, siRNA release ability, cytotoxicity, and cellular internalization in a human immortalized keratinocyte cell line (HaCaT) and a mouse embryo fibroblast cell line (NIH/3T3) were also evaluated. Furthermore, in vitro skin penetration and silencing efficiency studies were carried out.

## 2. Materials and Methods

### 2.1. Materials

Compritol^®^ 888 ATO (glyceryl behenate) was kindly supplied by Gattefossé (Paramount, NJ, USA) and Cremer Oleo GmbH & Co.KG (Hamburg, Germany). Tris(hydroxymethyl)aminomethane (Tris–HCl) was purchased from Merck KGaA (Darmstadt, Germany), and branched polyethylenimine (PEI) (25 kDa) and poloxamer 188 were purchased from Sigma Aldrich Co. (St. Louis, MO, USA). UltraPure™ Agarose was purchased from Invitrogen (Carlsbad, CA, USA). The heparin used in this study was obtained from Blausiegel (Cotia, SP, Brazil). Loading buffer (6× DNA loading dye #R0611) was acquired from Thermo Scientific (Rockford, IL, USA). Fetal bovine serum (FBS) and Opti-MEM™ media were obtained from Gibco (Grand Island, NY, USA), and Dulbecco’s modified Eagle’s medium (DMEM) and antibiotic-antimycotic cell culture solutions were obtained from Sigma Aldrich Co (St. Louis, MO, USA). Silencer™ Negative Control siRNA (siRNA), Silencer™ FAM (carboxyfluorescein)-labeled, and Alexa-fluor^®^ 647-labeled siRNA were purchased from Ambion^®^ (Austin, TX, USA). Mouse Fibroblast cells (BALB/NIH/3T3 strain clone A31 and HaCaT cells) were obtained from the Rio de Janeiro cell bank (Rio de Janeiro, RJ, Brazil). Lipofectamine^TM^ 2000 and Prolong™ Gold antifade reagent with DAPI were obtained from Life Technologies (Paisley, U.K.). The pCMV-Luc vector, which expresses Firefly luciferase (FL), was obtained from PlasmidFactory (Bielefeld, Germany). The pGL4.74 (hRluc/TK) vector, which expresses Renilla luciferase (RL), and Dual Luciferase were obtained from Sigma Aldrich (Darmstadt, Germany). The Reporter Assay System kit was acquired from Promega (Fitchburg, WI, USA).

### 2.2. SLPHNs Preparation

SLPHNs were prepared by hot emulsion high shear homogenization followed by sonication methods. Briefly, the lipid matrix containing Compritol^®^ 888 ATO and Tris–HCl buffer (100 mM, pH 6.5) as aqueous phase, PEI, and poloxamer 188 were heated separately to 75 °C. The mixture of aqueous phase and the oily phase was dispersed under high shear homogenization at 30,000 rpm for 2.5 min by Ultra-turrax (IKA T10 basic, Staufen, Germany) and then by ultrasonic processing for 5 min (Sonics VCX 750, Newtown, PA, USA, 13 mm probe and 40% amplitude). SLPHNs were formed after cooling the dispersion to 25 °C. In order to optimize the average size and polydispersity index (PdI), SLPHN samples were prepared using varying concentrations (%) of Compritol^®^ 888 ATO, PEI, and poloxamer.

### 2.3. Characterization of SLPHNs

#### 2.3.1. Particle Size, Zeta Potential, Nanoparticle Concentration, and Morphology

Nanoparticles were analyzed by dynamic light scattering (DLS) in NanoSize ZS (Malvern Instruments, Malvern, U.K.) using the DTS Nano software v5.03. The colloidal solutions were loaded into disposable plastic cuvettes and examined using backscattering light at a 90° angle. Size and distribution were measured in triplicate and expressed as Z-average diameter and PdI. The zeta potential of nanoparticles was also determined by observing their electrophoretic mobility in an electric field using the same equipment.

Nanoparticle tracking analysis (NTA) was determined using a NanoSight LM20 (NanoSight, Amesbury, U.K.). The samples were appropriately diluted and introduced into the sample chamber using sterile syringes. All measurements were carried out at room temperature.

The size and morphology of SLPHNs were determined by atomic force microscopy (AFM). Nanocarrier samples were prepared by depositing dilute particle dispersions on a freshly cleaved mica plate and drying with argon. Images were obtained using a Shimadzu Scanning Probe Microscope SPM-9600 model (Kyoto, Japan) equipped with a 100 μm tripod scanner and pyramidal cantilevers with silicon probes (force constant: 10–130 N/m) at a resonance frequency of 204–497 kHz. All measurements were performed in intermittent-contact mode at a scan speed of approximately 1 Hz to avoid damage to the sample surface.

#### 2.3.2. Physical Stability of SLPHNs

The physical stability of SLPHNs (*n* = 3) was assessed for 90 days under various conditions according to the Drug Stability FDA Guidelines [25]. These conditions included a low temperature of 4 °C and a high temperature of 30 °C with a relative humidity of approximately 75%. The evaluation of physical stability was based on monitoring the mean Z-average diameter and zeta potential of the formulations using DLS analysis.

### 2.4. Electrophoretic Mobility Shift Assay

#### 2.4.1. Evaluation of siRNA Binding and Polyanion Competition

The ability of siRNA to bind to SLPHNs was studied using an agarose gel assay [15,26]. For this, free siRNA and SLPHNs (1:4 *v*/*v*) with and without siRNA (10 µM) were prepared and incubated for 30 min. Electrophoresis was carried out in 2% agarose gel at 100 V for 20 min in Tris–acetate–EDTA (TAE) buffer (pH 8.0) after the addition of loading buffer (Bromophenol blue (0.25% *w*/*v*), xylene cyanol FF (0.25% *w*/*v*), Orange G (0.25% *w*/*v*), Tris–HCl pH 7.5 (10 mmol/L), ethylenediamine tetra-acetic acid (10 mmol/L), and saccharose (0.65% *w*/*v*)). The siRNA mobility shift was observed and photographed under UV light after addition of ethidium bromide (5 mg/mL) to the gel. Image acquisition was performed using the Quantity One software v.4.6.6.

To assess the stability of siRNA after decomplexation from SLPHNs, a polyanion competition assay was performed using heparin as a competitor [26,27]. Briefly, siRNAs–SLPHNs were prepared as previously described, and 10 µL of heparin (5000 IU/mL) was added. The samples were subjected to electrophoresis on agarose gels after incubation at 37 °C for 1 h using the same conditions described above.

#### 2.4.2. Serum Stability Study

The protective effect of SLPHNs against siRNA degradation was evaluated by incubating siRNA–SLPHN samples with 25% fetal bovine serum (FBS) for 24 h at 37 °C. Simultaneously, free siRNA and Lipofectamine™–siRNA samples were also incubated with 25% FBS at 37 °C for 24 h, serving as negative and positive controls, respectively. Following incubation, all samples were further treated with 10 µL of heparin (5000 IU/mL) for 1 h to ensure complete release of siRNA from the formulations [28]. The remaining percentage of intact siRNA was then analyzed using agarose gel electrophoresis as described in Section 2.4.1.

### 2.5. In Vitro Skin Penetration Study

In vitro permeation studies were conducted following the protocol outlined in OECD Guideline 428 [29]. Porcine ear skin was used as the biological membrane, and FAM-labeled siRNA [30] was employed in the study. Vertical Franz diffusion cells with a diffusion area of 0.68 cm^2^ were utilized. Each cell was filled with 3 mL of 100 mM phosphate buffer (pH 7.4 ± 0.2) at a temperature of 32 °C. The cells were maintained under continuous stirring at 400 rpm throughout the experiment. The dermatomized porcine ear skins (500 µm) were mounted in a Franz diffusion cell with SC facing the donor compartment, where 100 µL of the samples (naked siRNA–FAM and siRNA–FAM–SLPHN–0.25%) were applied. After 24 h of permeation, the skins were carefully removed from the Franz diffusion cell, and the surface was thoroughly washed with distilled water to eliminate any excess formulation. Subsequently, the skins were frozen in Tissue-Tek^®^ (Pelco International, Redding, CA, USA) embedding compound with acetone at −30 °C. A cryostat microtome (Leica, Wetzlar, Germany) was used to section the skin into 20 µm thick slices at a temperature of −20 °C, and slices were mounted on glass slides. The fluorescence intensity and depth of FAM-labeled siRNA within the skin were examined using fluorescence microscopy (Axioskop 2 plus, Carl Zeiss, Gottingen, Germany) along 640 nm and 730 nm band-pass excitation and emission filters. Images analysis was performed using the AxioVision software v.4. Due to skin auto-fluorescence, skin sections treated with PBS were used as a control. Consistent sensitivity and exposure settings were applied to all samples during the fluorescence microscopy analysis.

### 2.6. Cellular Studies

#### 2.6.1. Cell Culture Conditions

Non-tumorigenic keratinocytes (HaCaT) and murine fibroblasts (NIH/3T3) were individually cultured in Dulbelco’s modified Eagle’s medium (DMEM) rich in glucose supplemented with 10% heat-inactivated fetal bovine serum (FBS) and 1% (*v*/*v*) of an antibiotic solution containing 10,000 IU penicillin, 10 mg streptomycin, and 25 µg amphotericin B/mL. Cells were incubated at 37 °C in a humidified incubator (95% relative humidity) with 5% CO_2_ atmosphere.

#### 2.6.2. Cellular Viability

For the relative viability assay of HaCaT and NIH/3T3 cells, the resazurin assay was employed after 24 h of incubation. When the cells reached approximately 80% confluence, they were harvested from T-flasks using 0.05% (*v*/*v*) trypsin. Subsequently, 5 × 10^4^ cells/well for HaCaT cells and 1 × 10^4^ for NIH/3T3 cells were seeded onto 96-well plates and incubated at 37 °C and 5% CO_2_ atmosphere for 24 h to allow for cell attachment. Once the cells were attached, the culture medium was replaced with formulations containing SLPHN–0.15 PEI and SLPHN–0.25% PEI, along with various nanoparticle concentrations ranging from 1.59 × 10^10^ to 52.4 × 10^10^ particles/mL.

After an additional 24 h of incubation, the cells were washed with PBS, and resazurin solution (0.025 mg/mL) was added to each well. Following 4 h of incubation, fluorescence was measured using a Biotek plate reader (Synergy model) with an excitation wavelength set at 540 and emission at 590 nm. Untreated cells served as the negative control, and three repeated measurements were performed for each sample. The results were expressed as a percentage of cell viability.

#### 2.6.3. Cellular Internalization by Flow Cytometry

The cellular uptake of Alexa Fluor^®^ 647-labeled siRNA (siRNA Alexa Fluor^®^ 647) mediated by SLPHN was quantified by flow cytometry. HaCaT and NIH/3T3 cells were seeded onto 12-well tissue culture plates at a density of 3 × 10^5^ cells/well and 1.25 × 10^5^ cells/well, respectively. After 24 h, 100 µL of naked siRNA Alexa Fluor^®^ 647 (40 pmol/well) (free siRNA) or siRNA complexed with SLPHNs was added to the cells and incubated for 24 h at 37 °C with 5% CO_2_. After this period, cells were washed twice with PBS buffer and trypsinized, and cell uptake was assessed by flow cytometry using a Facscanto BD FACSCalibur™ Cytometer equipped with 3 lasers (488, 633, and 405 nm) and a 660/10 nm emission filter for Alexa Fluor^®^ 647. Propidium iodide (50 µg/mL) was used as an indicator of cell viability. Untreated cells (siRNA control) and cells treated with Lipofectamine™–siRNA were used as controls; 10,000 events were collected per analyzed sample.

#### 2.6.4. Intracellular Localization of SLPHNs by Confocal Microscopy

In vitro cellular localization of siRNA–SLPHNs was investigated by confocal microscopy using a LEICA—TCS SP2 microscope (Leica, Heidelberg, Germany). For this, a siRNA-labeled Alexa Fluor^®^ 647 was used in the formulation samples (siRNA–SLPHNs), and naked siRNA (free siRNA) was used as a negative control. HaCaT and NIH/3T3 cells were seeded onto coverslips (previously treated with glutaraldehyde) at 3 × 10^5^ cells/well and 1.25 × 10^5^ cells/well, respectively, in 12-well plates to guarantee 60–80% confluence. After 24 h, 100 µL of the siRNA Alexa Fluor^®^ 647 (40 pmol/well) naked or complexed with SLPHNs was added to the cells and incubated for 24 h. Cells were washed with PBS twice and fixed overnight with a 1% formaldehyde solution in PBS. After fixation, cells were washed twice with PBS and mounted on glass cover slides using Prolong™ Gold containing the blue fluorescent stain 4’,6-diamidino-2-phenylindole (DAPI) for nucleus labeling. Cells treated with Lipofectamine™–siRNA were used as a positive control. Finally, confocal images of the cells were taken using a 63× oil-immersion objective under emission filters of 350/470 and 660/10 nm, suitable for DAPI and siRNA Alexa Fluor^®^ 647, respectively.

#### 2.6.5. In Vitro Silencing Efficiency of siRNA–SLPHNs

In the luciferase reporter assay, HaCaT cells (1 × 10^6^ cells) were transfected with reporter vectors containing Firefly luciferase (pCMV-Luc) and Renilla luciferase (pGL4.74 hRluc/TK) using Lipofectamine™ 2000 in antibiotic-free medium. After 24 h, transfected cells were seeded onto 96-well plates at a density of 5 × 10^4^ cells/well. Once cells reached approximately 70% confluence, they were treated with SLPHN (containing 0.15 and 0.25% PEI) or Lipofectamine™ 2000. Both complexed with 50 pmol of siRNA per well. The siRNA used targeted Firefly luciferase (siLuc) or nonspecific siRNA (ns-siRNA). Cells were then incubated for an additional 24 h, washed with PBS, and maintained in fresh medium for another 24 h at 37 °C in a 5% CO_2_ atmosphere.

Following incubation, cells were lysed with Passive Lysis Buffer (Promega) using 200 μL per well. To measure luciferase activity, 10 μL of cell lysates and 50 μL of Luciferase Assay Reagent were added to a white 96-well plate. The luminescence was immediately measured using a plate reader, with an initial mixing period of 2 s, followed by luminescence measurements at 0.5 s intervals for 5 s. Subsequently, 50 μL of Stop & Glo Reagent was added to each well, and luminescence measurements were repeated.

To obtain normalized data, the Firefly luciferase luminescence was divided by the Renilla luciferase luminescence using the formula (Firefly luminescence AUC 5 s/Renilla luminescence AUC 5 s) [28,31].

### 2.7. Statistics

Results are presented as mean ± standard error of the mean (SD). One-way analysis of variance (ANOVA) followed by Tukey’s multiple comparison test was used as statistical analysis to compare all groups studied. Significance levels were reported as * *p* < 0.05, ** *p* < 0.01, and *** *p* < 0.001. All statistical analyses were conducted using GraphPad Prism software v.8.4.

## 3. Results and Discussion

SLPHNs have been shown to be novel nanocarriers for topical and transdermal drug delivery. They have a dual advantage over other lipid nanoparticles due to their vesicular and particulate nature, the high biocompatibility of lipids [32,33], and the structural contribution of PEI molecules. This study demonstrated the successful preparation of SLPHNs and their effective utilization as nanocarriers for the delivery of siRNA to the skin.

### 3.1. Preparation and DLS Characterization of SLPHNs

#### 3.1.1. SLPHNs Preparation

A hot emulsion high shear homogenization followed by the sonication method was used to prepare the SLPHNs developed in this study. We selected Compritol^®^ 888 ATO, poloxamer 188, and PEI as SLPHN components due to their favorable characteristics for topical application and ease of formation of nanoscale delivery systems. PEI was incorporated into the systems to form complexes with nucleic acid by non-covalent bonds [34]. To assess the influence of each component of the SLPHNs on particle sizes and polydispersity index, 15 different samples were prepared with different concentrations of Compritol^®^ 888, PEI, and poloxamer 188 (Table 1).

#### 3.1.2. Effect of Compritol and Poloxamer on Nanoparticle Composition

First, the influence of different amounts of Compritol^®^ 888 (1 to 5%) on particle size and surface charge of the SLPHNs was evaluated. For that purpose, the amount of poloxamer 188 and PEI concentration were fixed at 1.5 and 1% (*w*/*w*) in the samples (F2 to F6). Particle size and polydispersity index (PdI) increased proportionally with the addition of Compritol^®^ 888, perhaps due to the macroscopically observed high viscosity of the lipid phase, which contributed to the increase in superficial tension, leading to the formation of larger nanoparticles. Ribeiro and coworkers reached a similar result when they used a factorial design to investigate the role of each excipient in the desired responses (size, zeta potential, and PdI) in natural lipid-based nanocarriers [35,36]. Our findings are also consistent with those of Yalcin et al. when the authors optimized gemcitabine hydrochloride-loaded LPHNs based on an experimental approach. The study employed PLGA, soya phosphatidylcholine, and DSE-PEG as independent variables (components). It was observed that as the lipid/polymer ratio increased from low to high levels, there was a noticeable upward trend in particle size values [37].

Compritol^®^ 888 is composed of different esters of behenic acid with glycerol (15–23% monoglycerides, 40–60% diglycerides, 21–35% triglycerides) [38]. This lipid composition is regarded as a safe and biocompatible excipient that is widely used for the preparation of lipid nanocarriers because of various advantages, such as sustained release, better lymphatic targeting, and chemical stability, resulting in a suitable ability to load lipophilic and/or hydrophilic drugs [38,39]. It is noteworthy that lipid carriers containing Compritol^®^ exhibit a platelet-like shape, which allows them to easily slide between corneocytes after penetrating the intercellular lipids of the skin. This unique shape provides these carriers with adhesiveness and promotes skin hydration properties [40]. In terms of nanocarrier characteristics, Wang et al. prepared LPHNs using six types of solid lipids and observed that glycerides showed superior colloidal properties in terms of smaller particle size and narrower size distribution than fatty acids [41].

Based on our findings (Table 1), 2% Compritol^®^ 888 was selected and then combined with poloxamer 188 at four concentrations (0.5, 1.5, 3, and 5% *w*/*w*, samples F7 to F9). Thus, particle size decreased with increasing surfactant concentration, possibly due to a reduction in interfacial tension [24,42]. Furthermore, it has been reported that the presence of a surfactant such as poloxamer increases particle stability by covering the particle surface and preventing particle agglomeration [43].

#### 3.1.3. Influence of Branched PEI-Coated Nanoparticles

The F3 formulation containing Compritol^®^ 888 (2% *w*/*w*) and Poloxamer 188 (1.5% *w*/*w*) was selected for evaluation of PEI influence on particle size and PdI. Particle size and PdI of samples F10 to F15 increased with increasing PEI concentration. Similar results were previously reported when PEI was incorporated on both PLGA (poly (lactic-acid coglycol)) and PEG (polyethylene glycol)-based nanoparticles [44,45].

Furthermore, the zeta potential of the nanoparticles changed from negative in the particles without PEI (F1) to positive in the SLPHNs (F2 to F15). This positive surface charge of the particles is a result of the alignment of the PEI on the nanoparticle surface. The positively charged particle surface can facilitate particle adherence to negatively charged cellular membranes. This interaction promotes and enhances intracellular uptake of the particles [18,46]. Taking this into account, the SLPHNs consisting of Compritol^®^ 888 (2% *w*/*w*), poloxamer 188 (1.5% *w*/*w*), and PEI concentrations of 0.15% (F11) and 0.25% *w*/*w* (F13) were selected for further physicochemical characterization and in vitro studies.

### 3.2. Nanoparticle Tracking Analysis (NTA) and Morphology of SLPHN

Increasing the PEI concentration from 0.15% to 0.25% had no influence on particle concentration, which was similar for SLPHNs–0.15% PEI and SLPHNs–0.25% PEI (2.54 × 10^13^ to 2.62 × 10^13^ particles/mL, respectively). Also, a non-significant difference was observed between particle size determined by NTA and that determined by DLS. These were relevant parameters and important findings for the next in vitro studies since the recommended metric is the number of particles/volume.

**Figure 1:**
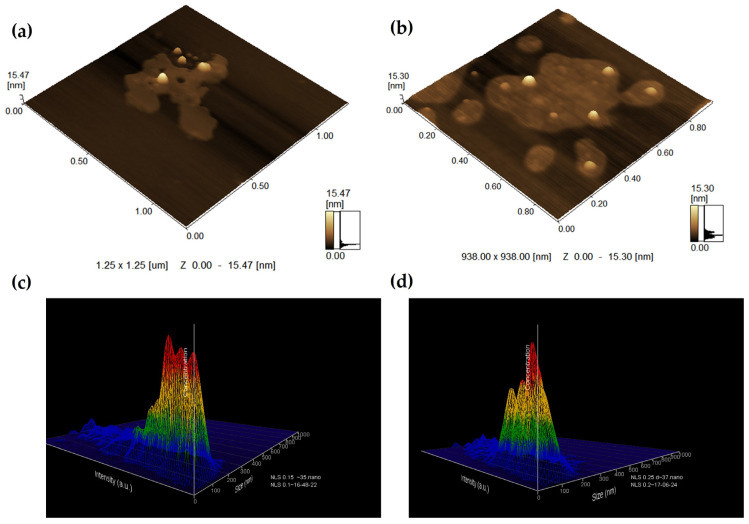
3D size distribution profile obtained by AFM (**a**,**b**) and NTA 3D (**c**,**d**) topographic image of SLPHN 0.15% PEI (**a**,**c**) and SLPHN 0.25% PEI (**b**,**d**).

As expected, the AFM images showed spherical particles with smooth surfaces (Figure 1). The chemically homogeneous lipid allows for the formation of perfect crystals that have a well-defined platelet-like pattern of the β-modification. Spherical lipid nanoparticles can be obtained with a combination of heterogeneous surfactants and heterogeneous lipids [47,48]. The morphology of nanoparticles is typically influenced by the composition of the lipid matrix and can manifest as a spheroidal, anisometric, or flat shape [46]. The specific shape that the nanoparticle assume is determined by both their size and the polymorphic shape of the lipid used in the formulation [43].

The average diameter of 58 nm and 67.51 nm determined by AFM for SLPHNs–0.15% PEI and SLPHNs–0.25% PEI, respectively, is smaller than the diameter values determined by DLS and NTA, which were in the order of 170 nm approximately. In general, this difference is related to the principles of each technique. On the one hand, the size values obtained by the DLS and NTA techniques are calculated using the Stokes–Einstein equation, which considers the hydrodynamic diameter of the particles composed by the particle diameter and its stabilization layer formed by the presence of the surfactant used [49]. Indeed, NTA is a technique that provides information about the concentration of nanoparticles and measures their size based on the analysis of light scattering and Brownian motion of individual nanoparticles [47,50]. With AFM, on the other hand, the microscope allows for the measurement of the diameter of nanoparticles without the stabilization layer [51].

### 3.3. Physical Stability Studies

**Figure 2:**
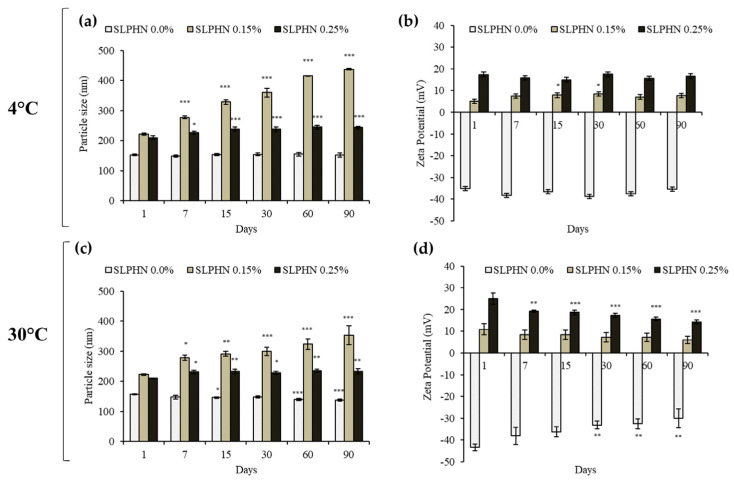
Physical stability data based on particle size (nm) (**a**,**c**) and zeta potential (mV) (**b**,**d**) of SLPHNs in the absence (SLPHN 0.0) and presence of PEI (SLPHN 0.15 and SLPHN 0.25) over 90 days at 4 °C (top, **a**,**b**) and 30 °C (bottom, **c**,**d**). * *p* < 0.05; ** *p* < 0.01; *** *p* < 0.001 compared to day 1 of each formulation. One-way ANOVA (95% confidence interval) and Tukey’s test.

Monitoring changes in zeta potential, particle size, appearance, and viscosity over an extended storage period allows assessment of the physical properties of lipid nanoparticles. External parameters such as temperature appear to be of primary importance for long-term stability [23]. Here, the physical stability properties of SLPHN–0.0%, –0.15%, and –0.25% PEI were assessed over a period of 90 days. When particle sizes were compared, SLPHN–0.25% PEI samples exhibited physical stability at both low temperatures (4 °C) and accelerated conditions (30 °C), as shown in Figure 2a,c. When lower amounts of PEI (0.15%) were inserted into the SLPHN, a progressive increase in particle size was observed throughout the experimental period, demonstrating the instability of the samples in these experimental conditions. These results could be related to the amount of PEI molecules on the nanoparticles, which presumably changes the structural conformation of the nanoparticles over the days. We believe that a PEI of 0.25% favors the stability of the formulation by a combination of steric hindrance and electrostatic repulsion and is, therefore, more stable over long periods than SLPHNs with less PEI. Also, the changes in the zeta potential of the SLPHNs–0.25% PEI were not significant over 90 days under experimental conditions (Figure 2b,d). Previously, our research group has demonstrated that polymers such as PEI can also act as a stabilizing agent in lipid-based nanoparticle systems and modify the surface charge [12,14,15,52].

Another contribution to the long-term stability of solid lipid nanoparticles is the presence of poloxamer 188, which, because of its nonionic nature, stabilizes the nanoparticles by forming a coat on their surfaces [53]. Therefore, we attributed the physical stability of the SLPHN observed in this work to the SLPHN composition (lipid, polymer, and surfactant) and their relative ratios.

### 3.4. siRNA Binding and Stability of the SLPHNs–siRNA Complex

**Figure 3:**
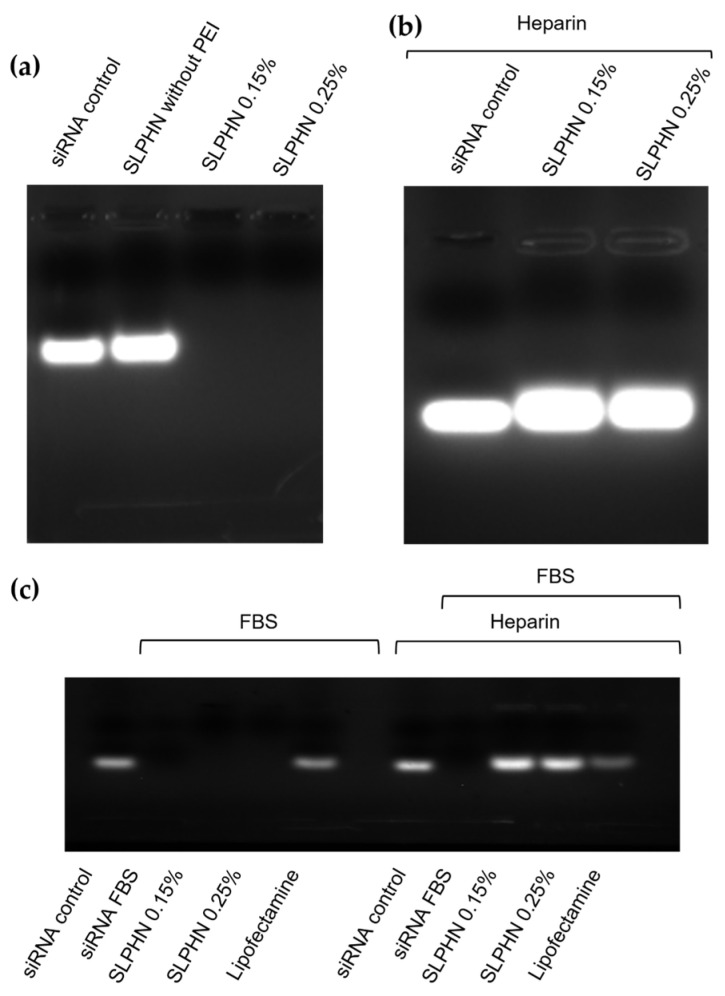
Electrophoretic mobility of siRNA after complexation with SLPHN without PEI, SLPHN with 0.15% PEI, and SLPHN with 0.25% PEI in the absence of heparin (**a**) and in the presence of heparin (**b**) and evaluation of the stability of siRNA under serum conditions (**c**). The siRNA control refers to free siRNA without FBS treatment, and siRNA-FBS refers to free siRNA after FBS treatment.

Figure 3 summarizes the ability of SLPHNs to complex siRNA and the consequent inhibition of its electrophoretic migration. In contrast, the siRNA Control (free siRNA), which has a negative charge, migrated through the gel, whereas positively charged complexes remained at the top of the gel (SLPHN 0.15% PEI and SLPHN 0.25% PEI). The presence of PEI in SLPHNs was crucial to complex siRNA, as SLPHNs lacking PEI did not form complexes with siRNA (Figure 3a). Complex formation leads to the electroneutralization of the negative charge of siRNA, which is interesting for stronger cellular internalization and endosomal escape. The complexation of siRNA by PEI has been previously demonstrated in studies using liquid crystalline systems [12,14,15,26,52], providing a positive surface charge to nanoparticles and thus promoting electrostatic interactions between nanoparticles and siRNA.

After determining that SLPHNs formulated with PEI successfully complexed siRNA, heparin (5000 IU/mL) was added to the complexes, and a polyanion competition between siRNA and heparin for PEI amine groups was observed, suggesting dissociation of the siRNA–SLPHNs complex (Figure 3b). Generally, for effective delivery of intact siRNA, it is important that the complexes remain stable during the delivery process. However, it is also necessary for siRNA to be released into the cytosol to achieve gene silencing [54]. Polyanions, such as heparin, can disassemble gene complexes, facilitating the release of siRNA from the complexes [55]. In this study, the siRNA released from SLPHNs demonstrated a similar migration distance as the siRNA control, indicating that SLPHNs can readily release the siRNA without causing its degradation. This finding confirms the successful preservation of siRNA integrity during the release process from SLPHNs.

### 3.5. Protection of siRNA in SLPHN from Serum Degradation

The ability to resist nuclease degradation is crucial for successful siRNA delivery, both in vitro and especially in vivo [1,56,57]. In addition to us, several authors have successfully submitted their developed nanocarriers to 50% FBS to mimic serum conditions [58]. Furthermore, evaluation of the interplay between SLPHNs and serum proteins can provide important guidelines for predicting their performance in biological systems [59].

After a 24 h exposure to serum (Figure 3c), free siRNA (10 μM) was not stable in the presence of 25% FBS and underwent complete degradation. This finding highlights the susceptibility of free siRNA to degradation in the presence of serum, emphasizing the need for protective delivery systems to ensure its stability and effectiveness. In contrast, the siRNA complexed with SLPHNs or Lipofectamine™ was released intact after the heparin polyanion assay, proving that SLPHNs protect siRNA from serum degradation.

### 3.6. In Vitro Skin Penetration Study

Next, we evaluated the delivery of siRNA into the skin by the SLPHNs. The penetration of siRNA into the skin is challenging because of its negative charge and high molecular weight. Additionally, the SC, which serves as a highly efficient barrier, controls the penetration of molecules and microorganisms through the skin [60]. To overcome this barrier and facilitate the delivery of siRNA into the deeper layers of the skin, nanoparticles have been extensively investigated as topical or transdermal formulations [2,61].

**Figure 4:**
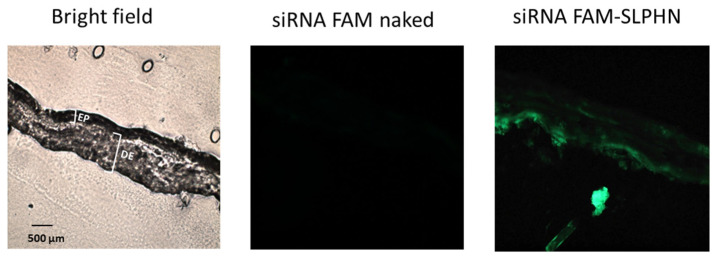
Distribution of labeled siRNA under light microscopy of in vitro penetration studies 24 h after application of naked FAM-siRNA (free siRNA) and FAM-siRNA–SLPHN–0.25% PEI (complexed siRNA) to untreated skin. The microscopic sections were visualized (Axioskop 2 plus microscope) at 640 nm and 730 nm band-pass excitation and emission filters, respectively, through a 10× objective. The formulation baths were tested in triplicate, and representative images are shown. EP: epidermis including stratum corneum, DE: dermis.

The in vitro siRNA penetration of SLPHN–0.25% PEI was evaluated using vertical diffusion cells and porcine ear skin as a biological membrane, which is a suitable alternative when barrier integrity is assured [30]. Figure 4 shows that naked siRNA displayed weak or no fluorescence on the skin surface. However, when the skin was treated with siRNA–SLPHN 0.25% PEI, a prominent green fluorescence signal was observed throughout the tissue, including the viable epidermis and dermis, indicating the presence of fluorescein-labeled siRNA. This observation suggests that the siRNA–SLPHN formulation allowed effective transport of siRNA across the stratum corneum, enabling the genetic material to be retained throughout the skin.

According to this finding, the enhanced penetration of siRNA into the deeper skin layers facilitated by SLPHNs is due to the combination of two factors. First, the positive charge of SLPHN increases the interaction of this nanocarrier with the skin surface, and second, the occlusion caused by solid lipid nanoparticles on the stratum corneum surface increases its permeability [62]. The interaction of these two factors may have further contributed to weakening the skin barrier and thus enhanced the skin penetration of siRNA.

### 3.7. Cellular Studies

#### 3.7.1. Cell Viability Study

The cell viability of SLPHNs was evaluated by the resazurin reduction assay to verify their action on representative skin cells (keratinocytes (HaCaT) and fibroblasts cells (NIH/3T3)) to determine the appropriate nanoparticle concentration for the next in vitro studies. The concentration of nanoparticles used in this experiment was determined from the NTA results (described in Section 3.1.1), assuming that the exposure dose of the particles, expressed in number per volume, is the best dose measure [63].

**Figure 5:**
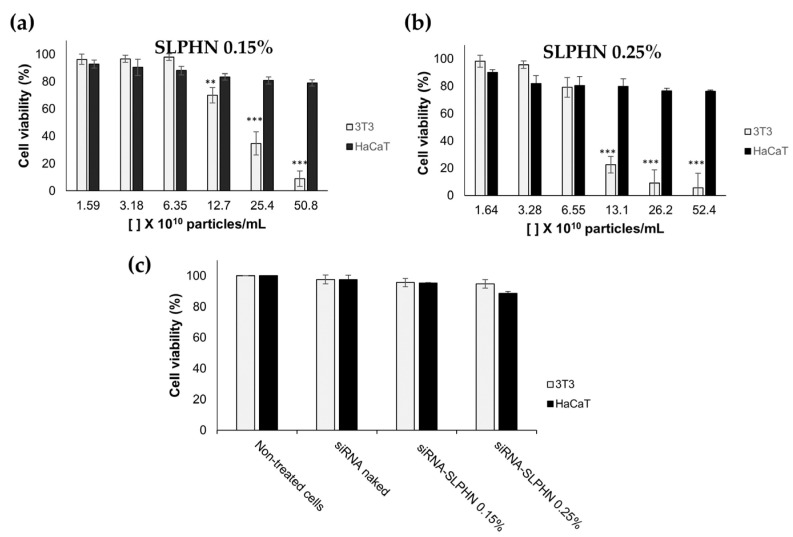
Cell viability of NIH/3T3 and HaCaT cells treated with different concentrations of SLPHN 0.15% PEI (**a**) and SLPHN 0.25% PEI (**b**) and cell viability of naked siRNA, siRNA–SLPHN–0.15% PEI and siRNA–SLPHN–0.25% PEI in NIH/3T3 and HaCaT cells (**c**). One-way ANOVA followed by Tukey’s post-test was used as statistical analysis. ** *p* < 0.01; *** *p* < 0.001 compared to the lowest particle concentration (× 10^10^ particles/mL) in each group.

Figure 5 shows the potential cytotoxicity of SLPHNs–0.15 PEI and SLPHNs–0.25% PEI after 24 h of treatment in HaCaT and NIH/3T3 cells. Overall, total lipid content or PEI concentration in SLPHNs applied to NIH/3T3 cells promoted a crucial toxicity factor causing cell toxicity. However, when SLPHNs were applied in a series of dilutions, this effect was less prominent, suggesting a dose-dependent cytotoxicity of SLPHNs.

In fact, all SLPHNs tested on HaCaT did not present a cytotoxic effect, but a cytotoxic effect on NIH/3T3 cells was observed with 25.4 × 10^10^ particles/mL for SLPHN–0.15% PEI and 13.1 × 10^10^ particles/mL for SLPHN–0.25% PEI (Figure 5a,b). These results suggest that NIH/3T3 cells were more susceptible to both the physical effect of sedimentation of low-density nanoparticles on the cell membrane and the chemical effect of their composition [64] compared with HaCaT cells, mainly because PEI can be cytotoxic at certain concentrations [26]. These findings are consistent with the findings of Lee and coworkers (2002) regarding phototoxicity [65]. Finally, cell viability above 80% was achieved in HaCaT and NIH/3T3 cells when using SLPHNs at approximately 6 × 10^10^ and 3 × 10^10^ particles/mL, respectively (Figure 5c). Under these conditions, both SLPHN formulations did not present cytotoxicity when complexed with siRNA.

#### 3.7.2. Cellular Uptake

**Figure 6:**
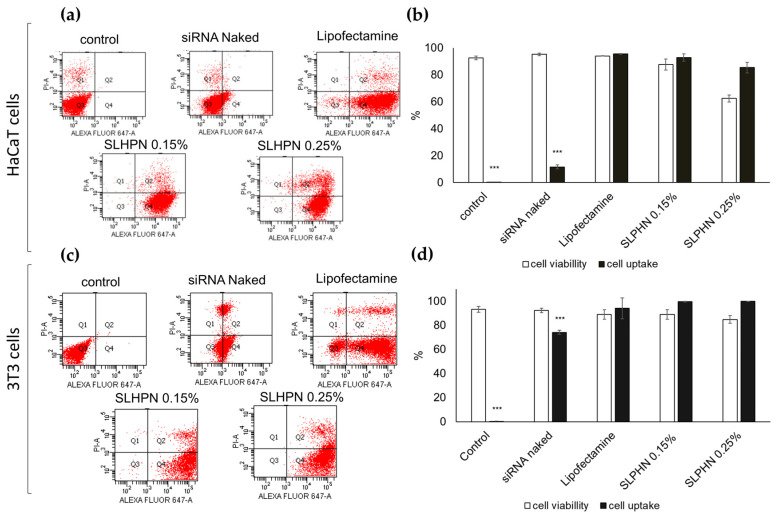
Representative plot obtained by flow cytometry (**a**,**c**) and cellular uptake/viability (**b**,**d**) in total cells using HaCaT and NIH/3T3 cells and siRNA Alexa Fluor^®^ 647 at a concentration of 40 pmol/well. Results represent the mean ± SD (*n* = 3). One-way ANOVA followed by Tukey’s post-test was used as statistical analysis. *** *p* < 0.001 compared with the Lipofectamine group.

The ability of SLPHNs to promote cellular uptake of siRNA was assessed by quantitative flow cytometry, which quantifies siRNA Alexa Fluor^®^ 647 within the cell after incubation of HaCaT and NIH/3T3 cells with different preparations. Figure 6 shows that siRNA cell internalization is above 85% for all tested SLPHNs, regardless of the cell line. For instance, the percentage of cells containing siRNA Alexa Fluor^®^ 647 was 92.93 ± 2.71% for SLPHN–0.15% and 85.37 ± 3.94% for SLPHN–0.25% in HaCaT cells (Figure 6b) and 99.70 ± 0.20% for SLPHN–0.15% and 99.93 ± 0.06% for SLPHN–0.25% in NIH/3T3 cells (Figure 6d). Notably, naked siRNA did not have a significant internalization (*p* < 0.001).

In fact, the strategy of releasing siRNA from SLPHN–0.15% PEI, SLPHNs–0.25% PEI, and Lipofectamine™ resulted in 5-fold higher fluorescence (*p* < 0.001) compared with naked siRNA. These findings suggest that the developed SLPHNs associated with PEI promote cell internalization through endocytosis due to their opposite charge to the cell membrane [55], allowing transfection of siRNA, a molecule that has limited cell penetration due to its hydrophilicity, high molecular weight, and negative charge [66,67].

#### 3.7.3. Intracellular Localization by Confocal Microscopy

**Figure 7:**
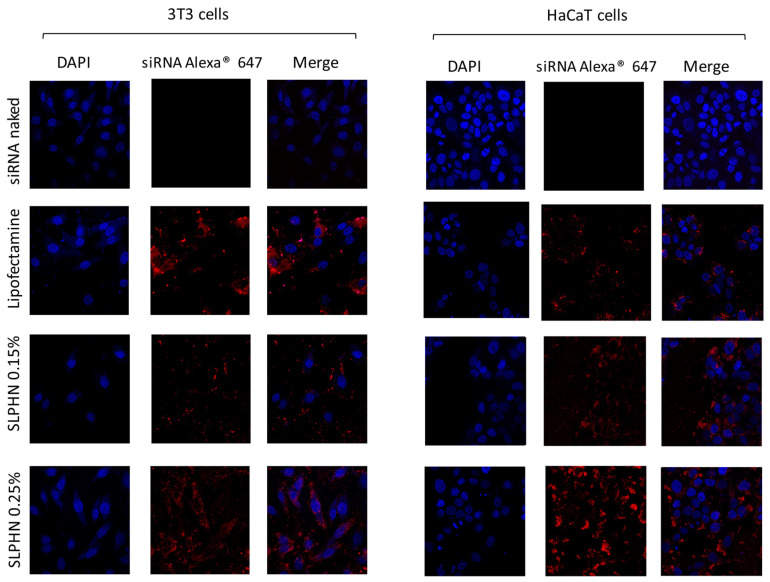
Cellular transfection in NIH/3T3 and HaCaT cells treated with free siRNA labeled with Alexa Fluor^®^ 647, Lipofectamine™, and SLPHNs. The cell nucleus was stained with DAPI (blue), and siRNA Alexa^®^ complexed with the internalized nanostructures was stained red.

The intracellular localization of siRNA–SLPHN in HaCaT and NIH/3T3 cells was performed by confocal microscopy. siRNA–SLPHNs were efficiently internalized by the cells and showed cytoplasmic localization (Figure 7). In this study, we used the siRNA–Lipofectamine^TM^ 2000^®^ as a positive control of transfection [14].

The presence and distribution of siRNA Alexa^®^ 647 red fluorescence around the DAPI-stained nucleus (blue) can be visualized in the merged images (Figure 7), evidencing the cell-wide distribution of siRNA–SLPHNs and siRNA–Lipofectamine™ in the cytoplasm of both NIH/3T3 and HaCaT cells. Stronger red fluorescence intensity was observed for siRNA Alexa^®^ 647 in keratinocyte cells (HaCaT) compared to fibroblast cells (NIH/3T3) in all tested samples. On the other hand, naked Alexa^®^ 647 siRNA exhibited minimal red fluorescence, indicating that there was no significant cellular uptake of free siRNA.

#### 3.7.4. In Vitro Silencing Efficiency of siRNA–SLPHN

The in vitro silencing study was performed in HaCaT cells stably expressing Firefly luciferase. The Renilla luciferase assay is commonly used to monitor transfection and silencing efficiency [28,31,68]. The silencing of genes expressing luciferase is measured by the ability of luciferase to convert chemical energy to light energy by catalyzing biological reactions using luciferin as a substrate. Thus, firefly luciferase activity in cells treated with Firefly luciferase-specific siRNA (siLuc) and nonspecific siRNA (ns-siRNA) was normalized by Renilla luciferase activity.

**Figure 8:**
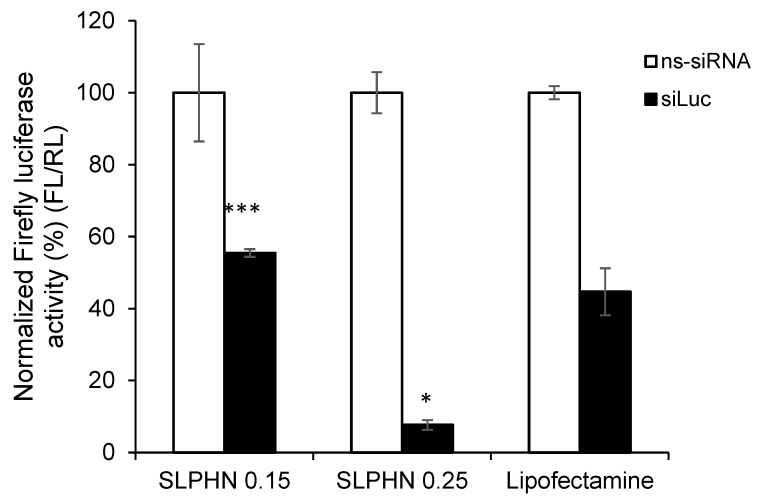
Percentage of inhibition of Firefly luciferase activity normalized by Renilla luciferase after treatment of HaCaT cells with siLuc (siRNA luciferase) and ns-siRNA (nonspecific siRNA) (100 pmol/1 × 10^5^ cells) complexed with SLPHNs (0.15% and 0.25% PEI) and Lipofectamine™. *** *p* < 0.001 and * *p* < 0.05 compared to cells treated with siLuc–Lipofectamine™ complex (100 pmol/1 × 10^5^ cells), (*n* = 3).

As demonstrated in the previous section, naked siRNA is unable to penetrate the cell membrane and reach the cytoplasm of HaCaT cells, so this sample was not used in this study. Figure 8 summarizes the silencing effect of anti-luciferase siRNA complexed with SLPHN and Lipofectamine™. All tested SLPHN samples demonstrated effective delivery of siLuc to HaCaT cells. Moreover, these formulations exhibited a significant reduction in Firefly luciferase expression in comparison to cells that were treated with ns-siRNA (*p* < 0.05).

Cells treated with siLuc–SLPHN and 0.25% PEI showed a 1.67-fold and 2.06-fold reduction in Firefly luciferase expression than Lipofectamine™ and SLPHN–0.15% PEI, respectively. SLPHN–0.25% PEI has a greater cationic charge, as shown by the zeta potential (Table 1), which allows a greater amount of siRNA to be complexed and increases cellular uptake (Figure 7). It is importante also to consider the contribution of the PEI interaction with particle components, mainly due to its interaction with poloxamer, being part of its constitution. The interaction of PEI with the poloxamer molecule was previously described [69], suggesting an interaction between protonated PEI amines and free electrons of the oxygen atoms of poloxamer.

All these characteristics resulted in greater silencing efficiency. By measuring luciferase activity, it is possible to determine whether the therapeutic genes carried by the nanoparticles are successfully delivered and expressed in the target cells, providing quantitative information about treatment efficacy. Therefore, we believe that before selecting a suitable inflammatory gene target, an appropriate luciferase assay must be conducted as a proof-of-concept silencing experiment.

Considering the data obtained in this work, we believe that the SLPHNs we developed stand out compared to several other lipid-based nanoparticles carrying siRNA for topical delivery [2] because of their favorable features, such as the biocompatibility of the components. The simple and reproducible preparation method favors the transfer of the production of these nanoparticles from the laboratory scale to the industrial scale. To our knowledge, this is the first time SLPHNs composed of Compritol^®^/PEI/poloxamer for topical delivery of nucleic acids have been reported, and they were found to be highly effective in complexing with siRNA and facilitating its release into the cytoplasm, leading to a therapeutic effect in vitro.

## 4. Conclusions

Novel and non-cytotoxic SLPHNs for siRNA delivery and topical application were successfully designed. The optimized SLPHN formulation exhibited a particle size of less than 200 nm and positive zeta potential. These particles remained stable when stored at 4 °C for a period of 90 days. This delivery system efficiently bound siRNA and showed high cellular transfection and cytoplasmic localization. Furthermore, the siRNA–SLPHNs released the specific siRNAs, reduced luciferase expression in HaCaT cells, and demonstrated significant knockdown of Firefly luciferase reporter expression. Moreover, the in vitro penetration assay demonstrated that SLPHN favored the penetration of siRNA into the epidermis, where several pathological processes of skin diseases occur.

Our findings demonstrate that the developed SLPHN–0.25% PEI is a promising nanoplatform for the cutaneous delivery of siRNA as it favored skin penetration of siRNA through the SC barrier and kept it in the epidermis, which is the desirable skin layer for topical application. The system also prevents transdermal delivery of siRNA, ensuring the safety of SLPHN as a carrier system of siRNA for topical delivery. In addition, SLPHNs–0.25% PEI formed stable siRNA complexes that efficiently transfected cells and promoted gene knockdown in vitro.

## Figures and Tables

**Table 1:** Physicochemical characteristics of SLPHNs in different compositions (*n* = 3).

Samples	PEI %	Compritol^®^ 888 ATO %	Poloxamer 188%	Aqueous Phase %	Size ± SD (nm)	PdI ^1^ ± SD ^3^	ZP ^2^ ± SD ^3^ (mV)
F1	0.00	2.00	1.50	96.50	143.20 ± 20.79	0.34 ± 0.03	−8.33 ± 7.88
F2	1.00	1.00	1.50	96.50	182.80 ± 13.45	0.37 ± 0.03	38.75 ± 2.14
F3	1.00	2.00	1.50	95.50	219.35 ± 8.84	0.42 ± 0.00	36.60 ± 0.28
F4	1.00	3.00	1.50	94.50	236.35 ± 14.24	0.43 ± 0.09	36.83 ± 1.56
F5	1.00	4.00	1.50	93.50	250.95 ± 59.25	0.45 ± 0.07	37.18 ± 1.74
F6	1.00	5.00	1.50	92.50	267.55 ± 43.18	0.44 ± 0.02	38.63 ± 3.21
F7	1.00	2.00	0.50	96.50	977.05 ± 67.81	0.39 ± 0.08	36.70 ± 0.85
F8	1.00	2.00	3.00	94.00	201.90 ± 0.42	0.36 ± 0.03	30.85 ± 0.78
F9	1.00	2.00	5.00	92.00	111.30 ± 15.41	0.37 ± 0.01	30.13 ± 0.66
F10	0.10	2.00	1.50	96.40	174.20 ± 1.68	0.23 ± 0.02	4.46 ± 0.75
F11	0.15	2.00	1.50	96.35	164.03 ± 4.74	0.27 ± 0.05	11.50 ± 1.15
F12	0.20	2.00	1.50	96.30	162.70 ± 11.01	0.38 ± 0.04	20.90 ± 2.87
F13	0.25	2.00	1.50	96.25	175.15 ± 17.71	0.29 ± 0.12	27.18 ± 1.55
F14	0.50	2.00	1.50	96.00	180.93 ± 5.85	0.48 ± 0.05	34.10 ± 3.55
F15	0.75	2.00	1.50	95.75	212.68 ± 15.85	0.46 ± 0.02	32.38 ± 3.53

^1^ PdI: polydispersity index; ^2^ ZP: zeta potential; ^3^ SD: standard deviation.

## Data Availability

Not applicable.
